# Importance of GluA1 Subunit-Containing AMPA Glutamate Receptors for Morphine State-Dependency

**DOI:** 10.1371/journal.pone.0038325

**Published:** 2012-05-31

**Authors:** Teemu Aitta-aho, Tommi P. Möykkynen, Anne E. Panhelainen, Olga Yu. Vekovischeva, Pia Bäckström, Esa R. Korpi

**Affiliations:** Institute of Biomedicine, Pharmacology, Biomedicum Helsinki, University of Helsinki, Helsinki, Finland; Tokai University, Japan

## Abstract

In state-dependency, information retrieval is most efficient when the animal is in the same state as it was during the information acquisition. State-dependency has been implicated in a variety of learning and memory processes, but its mechanisms remain to be resolved. Here, mice deficient in AMPA-type glutamate receptor GluA1 subunits were first conditioned to morphine (10 or 20 mg/kg s.c. during eight sessions over four days) using an unbiased procedure, followed by testing for conditioned place preference at morphine states that were the same as or different from the one the mice were conditioned to. In GluA1 wildtype littermate mice the same-state morphine dose produced the greatest expression of place preference, while in the knockout mice no place preference was then detected. Both wildtype and knockout mice expressed moderate morphine-induced place preference when not at the morphine state (saline treatment at the test); in this case, place preference was weaker than that in the same-state test in wildtype mice. No correlation between place preference scores and locomotor activity during testing was found. Additionally, as compared to the controls, the knockout mice showed unchanged sensitization to morphine, morphine drug discrimination and brain regional μ-opioid receptor signal transduction at the G-protein level. However, the knockout mice failed to show increased AMPA/NMDA receptor current ratios in the ventral tegmental area dopamine neurons of midbrain slices after a single injection of morphine (10 mg/kg, s.c., sliced prepared 24 h afterwards), in contrast to the wildtype mice. The results indicate impaired drug-induced state-dependency in GluA1 knockout mice, correlating with impaired opioid-induced glutamate receptor neuroplasticity.

## Introduction

In state-dependency, information retrieval is most efficient when the animal is in the same state as it was during the information acquisition. State-dependency has been implicated across species (human, [1]; rodent, [2]; *C. elegans*, [3]) and with various states [1], [4], including those induced by drugs [5]. Drugs induce an internal state (an affect) that can act as a cue in learning process: when an animal learns a behavior while intoxicated, it later better recalls the behavior while being again intoxicated. Drug-induced state-dependency might, thus, underlie behaviors that are driven by drug-associated cues, e.g. drug-induced relapses in addicted individuals [6], [7]. However, the molecular correlates of state-dependency remain to be resolved.

Glutamate receptors of α-amino-3-hydroxy-5-methyl-4-isoxazolepropionic acid (AMPA) type (for review, see [8]) possess a dual role in neuronal communication. First, they are mediators of fast-acting excitatory neurotransmission, and second, changes in their regulation constitute a molecular mechanism of neuronal adaptation underlying long-term potentiation, a putative cellular correlate of learning and memory [9]. More specifically, AMPA receptor GluA1 subunit-deficient mouse line (GluA1−/− mice) shows adaptation-dependent defects at neuronal circuitry [10] and behavioral [11] levels, suggesting that GluA1 subunit has a major role in neuronal adaptation.

GluA1 subunit has been proposed to play a role in the adaptation processes mediating the initial steps that eventually lead to compulsive drug use [12]. GluA1−/− mice show various phenotypes related to substance abuse as they i) fail to show cocaine-induced enhancement in AMPA/NMDA ratio in ventral tegmental area dopamine cells (VTA DA cells) [13], ii) display a specific impairment in stimulus reward learning by showing no conditioned reinforcement and abnormal second-order responding [14] and iii) express deficient morphine- and benzodiazepine-induced adaptation during development of tolerance and in withdrawal phase [15], [16].

Here, we studied drug-induced state-dependency using morphine as a state-forming stimulus in place conditioning setting. In GluA1−/− mice, the expression of morphine-conditioned place preference was tested with or without morphine to assess state-dependent testing conditions. We also studied the detection of morphine stimulus in drug discrimination paradigm, and morphine action in various brain regions using opioid-stimulated G-protein neurochemical assay. VTA DA neuronal plasticity induced by a single morphine injection was examined by electrophysiological *ex vivo* slice recordings. Our results suggest a role of AMPA receptors in morphine-induced state-dependency.

## Materials and Methods

### Ethics

All experimental procedures in this study were carried out with the approved permissions (ESAVI-0010026/041003/2010 and ESAVI-2010-05600/Ym-23) and had ethical approval from the State Provincial Government of Southern Finland. All efforts were made to minimize the number and suffering of animals.

### Animals

GluA1−/− mouse line was generated as described [10]. GluA1 line was backcrossed to C57BL/6J mouse line (Harlan BV., Horst, The Netherlands) at least 7 times prior to use. For the experiments, the GluA1−/− mice and the GluA1+/+ littermate controls were from heterozygous breedings. The animals were genotyped using PCR from tail-tip biopsy samples as described [10]. In the behavioral experiments, the animals were housed 2–4 animals to a cage from weaning, until used at the age of 2.5–4 months. All animals were naïve to pharmacological and handling manipulations until the start of the experiments. In the electrophysiological experiments, the animals were used at the time after weaning, at the age of 20–26 days. The animal facility had lights on from 6 a.m. until 6 p.m., temperature set at 20±1°C and relative humidity at 50±1%. Water and standard rodent food pellets (Harlan) were available *ad libitum*. All the experiments were performed during the light phase between 8 a.m. and 13 a.m. Altogether 156 GluA1+/+ (76 females, 80 males) and 129 GluA1−/− (60 females, 69 males) animals were used.

### Morphine injections

Morphine hydrochloride (Yliopiston apteekki, Helsinki, Finland) was daily dissolved in 0.9% NaCl solution (saline) and injected subcutaneously in a volume of 10 ml/kg, unless otherwise stated. Control animals were injected with equal volumes of saline.

### Conditioned place preference paradigm

Conditioning and place preference testing was performed in eight polycarbonate cages (45×22.5×15 cm; Tecniplast, Buguggiate, Italy) covered with transparent lids with ventilation holes. The cage floors were covered with either one of the two materials, which acted as conditioning stimuli: plastic material consisting of 1.2-cm-wide flat bars separated by 0.5 cm from each other and metal grid material of stainless steel wire mesh with 1-mm holes. These materials were selected from several different materials in 15-min preliminary pre-conditioning preference tests using a separate batch of naïve GluA1−/− and GluA1+/+ mice so that the animals showed no preference for either of them (data not shown). Locomotor activity (distance traveled in meters) and location of each mouse in place preference test (time spent in the morphine-associated side) was determined by Ethovision 3.0 video tracking system (Noldus Information Technology BV., Wageningen, The Netherlands) based on video image analysis of the center-point of the mouse. Between the trials all cages and floor materials were thoroughly washed with water and dried to remove the odors. The whole experiment was repeated 5 times using different animals with all groups being present in each sub-experiment, and the results were finally combined.

In the conditioning phase, two 30-min trials were performed per day on four consecutive days resulting in total of eight conditioning trials. Each day, in the first trial, saline injection was paired with either plastic or grid floor (CS− trial, conditioning stimulus with saline). In the second trial 4 h after the first one, morphine (10 or 20 mg/kg) was paired with the other floor type (CS+ trial, conditioning stimulus with morphine), so that half of the animals received morphine paired with plastic and the other half with grid. Immediately after the injections the mice were placed to the cages for 30 min. To ascertain that mice expressed no preference for either floor material, also control animals of both mouse lines were included, always receiving saline paired with both materials throughout the trials.

Place preference test was performed two days after the last conditioning trial at 10 a.m. The cage floor was covered with both plastic and grid materials, thereby bisecting it into two distinct equally-sized zones without any vertical borders between them. Thus, during both the place preference test trial and conditioning trials the area of the arena was identical, i.e. the animals had access to the whole apparatus during all phases of the experiment. Spatial orientation of the materials was balanced for the test groups. The animals were injected with saline or a priming dose of morphine (10 mg/kg), thus creating saline-state (Sal-state) or morphine 10 mg/kg-state (Mor10-state), respectively, and placed in the center of the cage. Locomotor activity and location of the mouse were observed for 15 min. Place preference was presented as time spent in the morphine-associated zone.

Locomotor activity of young 20- to 26-day-old GluA1−/− and GluA1+/+ mice was determined without injections for 30 min as reported above for the conditioning trial activity.

### Drug discrimination paradigm

Drug discrimination training was conducted in operant chambers (Med-Associates, Georgia, VT, USA) enclosed in ventilated sound-attenuating cubicles. The chambers were equipped with two retractable levers, an amber stimulus light above each lever, and a white houselight located on the opposite wall. The reinforcer was sweetened condensed milk (Törsleffs, Haugen –Gruppen Oy, Vantaa, Finland) diluted 1∶2 in water and delivered at a volume of 25 µl in a drinking cup located between the levers.

Mice were food restricted to an average 2.6 g of chow per day resulting to 85% of free feeding body weight for the duration of the experiment. Mice were trained to discriminate 5 mg/kg morphine from saline administered 20 min before start of the discrimination session. Twenty-min sessions were conducted Monday through Friday during the light period of the light-dark cycle, one session per day. Mice were initially trained to lever press for sweetened condensed milk after administration of 0 or 1 mg/kg morphine under a fixed ratio (FR) schedule 1. The morphine dose was gradually increased to 5 mg/kg and the response requirement to FR 5. This training procedure was adopted because of problems with nonexistent responding when training was started with the 5 mg/kg dose and FR 5 requirement. Morphine and saline were administered in a pseudorandom order and the positions of morphine and saline levers were counterbalanced among mice. Each training session started with the illumination of the houselight and the stimulus lights above the levers. Upon completion of the response requirement on the correct lever, the reinforcer was delivered and the houselight as well as the stimulus light above the correct lever were extinguished for 3.5 s.

Discrimination training between 0 and 5 mg/kg morphine under FR 5 schedule was considered stable when the following criteria were met for at least four out of five consecutive sessions: 1) ≥10 reinforcers earned per session, 2) first completed response requirement on the correct lever, and 3) ≥80% correct total responses. After these criteria were met, mice were tested with 0, 2.5, 5, and 10 mg/kg of morphine to generate a dose-response function. The morphine doses were administered in a within-subjects Latin square design. Each test session was preceded by two training sessions (one morphine, one saline session) satisfying the criteria stated above. If the criteria were not satisfied during these two sessions, animals were required to meet the criteria above for 4 out of 5 consecutive sessions before testing continued.

### Electrophysiology

Young mice (20–26 days old) were injected i.p. either with 10 mg/kg of morphine or saline. The brains were dissected out 20–30 h later and cut into slices, and whole-cell patch-clamp recordings of VTA dopamine neurons were conducted as previously described in [17]. In short, the horizontal slices containing the VTA were prepared in cutting solution containing (in mM): 60 NaCl, 2 KCl, 8 MgCl_2_, 0.3 CaCl_2_, 30 NaHCO_3_, 1.25 NaH_2_PO_4_, 140 sucrose, 10 D-glucose. Recordings were made in carbogen-bubbled artificial cerebrospinal fluid (ACSF, containing (in mM): 126 NaCl, 1.6 KCl, 1.2 NaH_2_PO_4_, 1.2 MgCl_2_, 2.5 CaCl_2_, 18 NaHCO_3_, and 11 D-glucose. ACSF was perfused over the slice at the rate of 2 ml/min. The drugs used in the recordings were diluted to the ACSF. Electrodes had a resistance of 3–5 MΩ when filled with internal solution containing (in mM): 130 cesium methanesulfonic acid, 10 HEPES, 0.5 EGTA, 8 NaCl, 5 QX314, 4 MgATP, 0.3 MgGTP, 10 BAPTA (pH 7.2–7.25), osmolarity 278±5 mOsm.

VTA in horizontal brain slices was recognized as an area medial to the substantia nigra compacta and medial to the terminal nucleus of the accessory optic tract. The neuron was identified as dopaminergic, if there was a clear hyperpolarization-activated cation-current (*I*_h_-current) seen after hyperpolarizing the cell in 10-mV steps to −130 mV [18]–[20].

Excitatory postsynaptic currents (EPSCs) were evoked by electrical pulses delivered at 0.1 Hz frequency (stimulator S-900, Cornerstone by Dagan) through a bipolar stimulus electrode. Picrotoxin (100 µM) was added to the ACSF to block GABA_A_ receptors. The recordings were performed in voltage clamp configuration at +40 mV (Axopatch 200B, Axon Instruments, Union City, CA, USA) [20]. The stable baseline EPSCs were recorded for 5–10 min. Then, 100 µM DL-AP5 was added to perfusion solution to block NMDA receptors, and after 5 min, the remaining current representing AMPA receptor-mediated current was recorded for 5–10 min with continued presence of DL-AP5. The recordings were filtered at 1 kHz and digitized at 20 kHz (Digidata 1322A, Axon Instruments). To calculate the AMPA/NMDA ratio, first the average AMPA receptor-mediated current was subtracted from the average baseline EPSC, revealing the NMDA receptor-mediated current, and then the AMPA/NMDA-ratio was calculated from the peak amplitudes of the currents. One to two cells (only one cell per slice) were studied per mouse. The recordings from different cells from the same animal were averaged and this average was used in the analysis. The drugs used in electrophysiology were purchased from Tocris Bioscience (Bristol, UK).

### μ-Opioid receptor agonist-stimulated GTPγ[^35^S] autoradiography

Guanosine 5′-(γ-[^35^S]thio)triphosphate (GTPγ[^35^S]) autoradiography was performed as described earlier [21] with minor modifications. GluA1+/+ and GluA1−/− mice were decapitated, their brains removed, and frozen on dry ice and stored at −80°C. Coronal sections of 14 µm were cut using a cryostat (Leica CM 3050 S, Leica Microsystems, Nussloch, Germany), thaw-mounted on Superfrost slides (Mentzel-Glaeser, Brauschweig, Germany) and stored at −80°C. Air-dried sections were surrounded by hydrophobic pen (Daido Sangyo Co., Tokyo, Japan). Sections were preincubated at 20°C for 20 min in 50 mM Tris-HCl (pH 7.4), 1 mM EDTA, 100 mM NaCl and 5 mM MgCl_2_ in a volume of 0.95 ml per slide. Thereafter, in the second preincubation, the sections were loaded with 2 mM guanosine diphosphate (GDP) in the same buffer for 60 min at 20°C in the presence of 1 µM adenosine A_1_ receptor blocker 8-cyclopentyl-1,3-dipropylxanthine (DPCPX, Tocris). DPCPX was added to reduce the extensive basal activity of adenosine receptors [22]. The final 90-min incubation with GTPγ[^35^S] (54–68 pM; Perkin Elmer, Boston, USA) took place in the buffer supplemented with 2 mM GDP, 1 mM dithiothreitol, 1 µM DPCPX and various concentrations of DAMGO (0.1–10 µM, Tyr-D-Ala-Gly-N-methyl-Phe-Gly-ol) or morphine (10 µM), in the presence and absence of 10 µM naltrexone. Nonspecific binding was defined in the presence of 10 µM unlabeled GTPγS. The sections were washed twice for 5 min at 0°C in 50 mM Tris-HCl (pH 7.4) and 5 mM MgCl_2_, and dipped in distilled water at 0°C, before drying under a fan at room temperature. The sections were exposed to film (Kodak BioMax MR, Eastman Kodak, Rochester, USA) at 8°C for 4 days together with [^14^C]-standards (GE Healthcare, Buckinghamshire, UK), and thereafter films were developed. Films were digitized and binding densities of the sections quantified using Dage MTI camera system (DAGE-MTI Inc., Michigan City, USA) combined with MCID Elite M5+ 4.0 software (Imaging Research Inc., St. Catharines, Canada) with [^14^C]-standards, which were used to convert optical density values to radioactivity values. Non-specific binding values were subtracted from all other values. DAMGO-stimulation data were fitted for EC_50_ and maximal stimulation (maximal DAMGO-induced stimulation expressed as a percentage of that observed with basal GTPγ[^35^S] binding) values by nonlinear regression analysis using GraphPad Prism 5.0 software (GraphPad Software, San Diego, CA, USA). The chemicals in the autoradiographic assay were from Sigma (St. Louis, MO, USA) unless otherwise stated.

### Statistics

Data were analyzed with either two- or three-way ANOVA with repeated measures when applicable, followed by either *post hoc* Bonferroni test (more than two groups) or by independent samples unpaired *t*-test (two groups). Correlations between locomotor activity and place preference during the place preference test were calculated using Pearson's correlation. Statistical analyses were performed by using the statistical software packages SPSS 15.0 for Windows (SPSS, Chicago, IL, USA) and the Prism 5.0 program (GraphPad Software). Statistical significance was set at *p*<0.05. Data from both sexes were pooled unless otherwise stated. Data are expressed as means ± standard errors of the mean (SEM).

## Results

### Locomotor activity in conditioning trials

**Figure 1:**
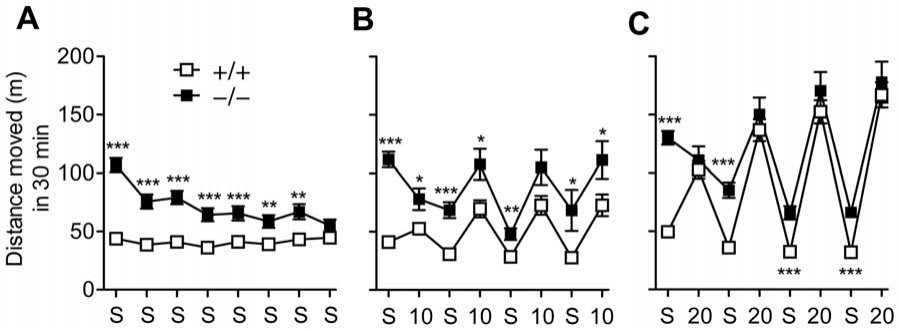
Distance moved during each of the eight conditioning trials for the GluA1−/− (−/−) mice and their littermate wildtype GluA1+/+ mice (+/+). In CS+ trials, animals were injected with saline (saline control animals) (**A**), morphine 10 mg/kg (**B**) or morphine 20 mg/kg (**C**) immediately before transferring them into cages, in which their locomotor activity was recorded for 30 min. Saline was injected in CS− trials. Data represent means ± SEM, *n* = 24–49. *p<0.05, **p<0.01, ***p<0.001, between the genotypes, *t*-test. S = Saline; 10 and 20 denote doses of morphine in mg/kg.

**Figure 2:**
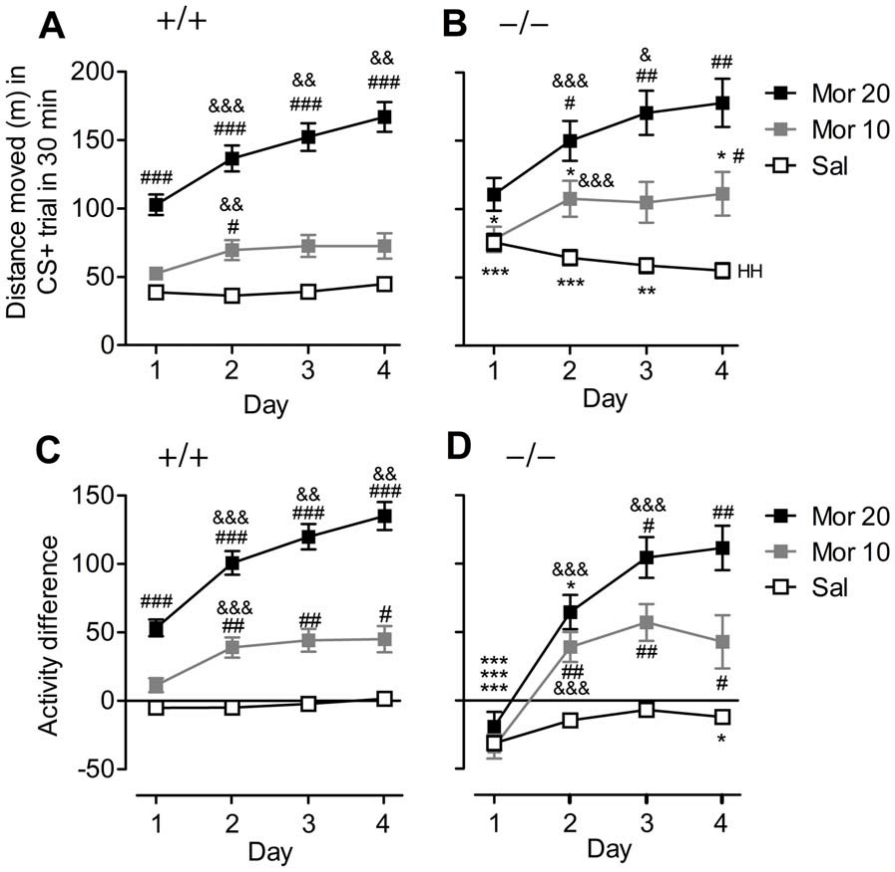
Morphine-induced locomotor sensitization in GluA1−/− and GluA1+/+ mice. (**A–B**), Distance moved in the CS+ (morphine, or saline for saline control animals) trials is shown. The scores indicate 1) significant morphine effects and dose-response (^#^ *p*<0.05, ^##^ *p*<0.01, ^###^ *p*<0.001, compared to the previous dose, Bonferroni test), 2) significant locomotor sensitization to morphine (^&^ *p*<0.05, ^&&^ *p*<0.01, ^&&&^ *p*<0.001, compared to the previous day, Bonferroni test), 3) significant habituation in GluA1—mice (saline control group) (^HH^ *p*<0.01, compared to the first trial, Bonferroni test). Data represent means ± SEM, *n* = 24–49. ^*^ *p*<0.05, ^**^ *p*<0.01, ^***^ *p*<0.001, between the genotypes, *t*-test. The scores were derived from the raw data depicted in Fig. 1. (**C–D**), The difference in distance moved between trials 1 and 2 (CS+ minus CS−) on each conditioning day. The data indicate 1) that the activity of GluA1−/− is strongly reduced, in comparison to GluA1+/+ mice, during the second trial of the first conditioning day after both saline control and morphine injections (^*^ *p*<0.05, ^**^ *p*<0.01, ^***^ *p*<0.001, between the genotypes, *t*-test), 2) that during the following days, GluA1−/− and GluA1+/+ mice were equally activated by 10 mg/kg morphine and 3) that morphine 20 mg/kg produced incremental locomotor activity in both mouse lines (^#^ *p*<0.05, ^##^ *p*<0.01, ^###^ *p*<0.001, compared to the previous dose, Bonferroni test). Data represent means ± SEM, *n* = 24–49. The scores were derived from the raw data depicted in Fig. 1.

Figure 1A–C represents an overview of the locomotor activities throughout the whole conditioning process in GluA1+/+ and GluA1−/− mice. GluA1−/− animals were hyperactive at the beginning of the eight-trial conditioning phase relative to GluA1+/+ animals (saline-treated control mice, genotype effect, F_1,50_ = 28.49, *p*<0.001), but habituated gradually to the level of GluA1+/+ mice (genotype×trial interaction, F_7,350_ = 18.15, *p*<0.001). No habituation was seen in GluA1+/+ mice (trial effect, *p*>0.05), with their locomotor activity remaining constant throughout the saline trials (see also Figs. 2A and 2C). No significant sex effects or interactions were found in place preference experiments (statistics not shown).

Figure 2A–B displays the effects of morphine and saline on locomotor activity during the four CS+ conditioning trials. Morphine increased locomotor activity in a dose-dependent manner in both genotypes (conditioning dose effect, F_2,170_ = 49.34, *p*<0.001). Morphine effect on locomotor activity was increased progressively after each conditioning trial (day effect, F_3,510_ = 41.00, *p*<0.001). The dose of 20 mg/kg produced a greater sensitization than the dose of 10 mg/kg (day×conditioning dose interaction, F_6,510_ = 27.90, *p*<0.001).

To take into account the different baseline activities of the GluA1+/+ and GluA1−/− mice, difference scores between each CS+ and corresponding CS− trials were calculated within mouse lines (Fig. 2C–D). In concordance with the data in Fig. 2A–B, morphine-induced activation was dose-dependent (conditioning dose effect, F_2,170_ = 61.02, *p*<0.001), and the animals sensitized to morphine (day effect, F_3,510_ = 131.73, *p*<0.001) in a dose-dependent manner (day×conditioning dose interaction, F_6,510_ = 30.10, *p*<0.001). However, the first morphine dose failed to activate the GluA1−/− mice (genotype effect and day×genotype interaction, F_1,170_ = 7.53, *p*<0.01 and F_3,510_ = 17.66, *p*<0.001, respectively).

### Place preference test

**Figure 3:**
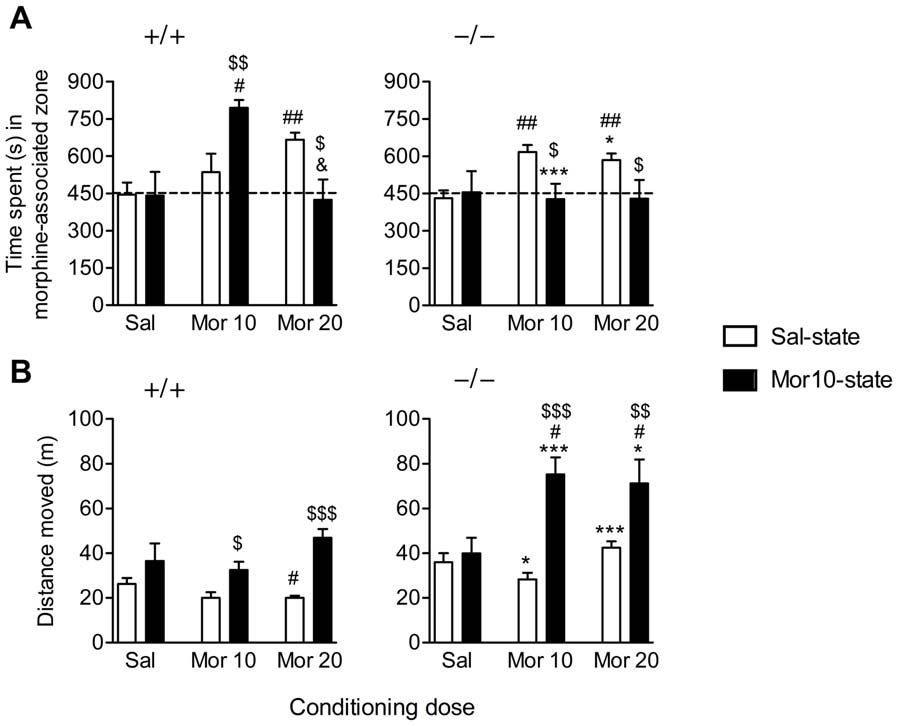
State-dependency is impaired in GluA1−/− mice in morphine-conditioned place preference. (**A**) Time spent in the morphine-associated zone during 15 min (900 s). Animals were tested either in Sal-state (priming injection of saline) or in Mor10-state (priming injection of 10 mg/kg morphine). (**B**) Distance moved during the 15 min preference test. Data represent means ± SEM, *n* = 8–33. ^*^ *p*<0.05, ^***^ *p*<0.001, between the genotypes, *t*-test; ^$^ *p*<0.05, ^$$^ *p*<0.01, ^$$$^ *p*<0.001, between testing states, *t*-test; ^#^ *p*<0.05, ^##^ *p*<0.01, between conditioning doses, compared to saline control, Bonferroni test; ^&^ *p*<0.05, between conditioning doses, compared to morphine 10 mg/kg conditioning dose, Bonferroni test. Sal = Saline, Mor = Morphine.

In the Sal-state, both GluA1+/+ and GluA1−/− mice expressed place preference that was dependent on the morphine conditioning dose (Fig. 3A; conditioning dose effect, F_2,158_ = 4.26, *p*<0.05). After the conditioning dose of 10 mg/kg, GluA1+/+ mice did not display place preference in the Sal-state (*p* = 0.3), whereas GluA1−/− mice showed a significant preference (however, no significant genotype difference here). After the conditioning dose of 20 mg/kg, slightly less place preference was observed in GluA1−/− mice compared to GluA1+/+ mice (*p*<0.05, *t*-test). In the Mor10-state, the place preference was either increased or decreased depending on the conditioning dose (conditioning dose×testing state interaction, F_2,158_ = 3.82, *p*<0.05). In GluA1+/+ mice, testing in Mor10-state increased place preference as compared to testing in Sal-state in mice conditioned with 10 mg/kg morphine, but decreased the preference in mice conditioned with 20 mg/kg morphine. However, GluA1−/− mice expressed no place preference when tested in Mor10-state independent of the conditioning dose (10 and 20 mg/kg) (genotype×conditioning dose×testing state interaction, F_2,158_ = 5.40, *p*<0.01).

In Sal-state, locomotor activity during the preference test was at higher level in GluA1−/− mice than GluA1+/+ mice (Fig. 3B; genotype effect, F_1,158_ = 49.00, *p*<0.001), indicative of the phenotypical hyperactivity of the GluA1−/− mice. However, no difference was detected between those groups that had received saline during all conditioning trials (*t*-test, *p*>0.05), indicative of complete habituation in the GluA1−/− mice. In GluA1+/+ mice in the Sal-state, locomotor activity decreased in the animals conditioned with 20 mg/kg morphine (conditioning dose effect, F_2,158_ = 5.07, *p*<0.01), but no such difference was detected in GluA1−/− mice (genotype×conditioning dose interaction, F_2,158_ = 4.22, *p*<0.05). Testing in Mor10-state increased locomotor activity in both mouse lines as compared to testing in Sal-state (testing state effect, F_1,158_ = 68.11, *p*<0.001). This increase in locomotor activity also was dependent on genotype and conditioning dose (genotype×conditioning dose×testing state interaction, F_2,158_ = 3.63, *p*<0.05): no increase was observed in mice that had received saline in all conditioning trials, but the morphine-conditioned GluA1−/− mice moved more than the corresponding GluA1+/+ mice.

Because alterations in locomotor activity during the place preference testing trials could possibly affect the expression of place preference [23], correlations between the place preference scores and the locomotor activities during the tests were determined. For both GluA1−/− and GluA1+/+ mice, Pearson correlation coefficients were calculated for each conditioning dose in two ways: first, separately for the groups tested in Sal-state and Mor10-state, and second, by pooling both state groups. In both cases the highest correlation score found was 0.43 (*p*>0.05), and thus in neither case were the correlations significant between the preference scores and locomotor activities (*p*>0.05, data not shown).

### Morphine discrimination

Using the drug discrimination paradigm, we aimed to compare the detection of internal opioid signals in GluA1−/− and GluA1+/+ mice. In discrimination training, both genotypes equally learned to discriminate morphine during the 40 training sessions (genotype effect, F_1,12_ = 0.15, *p*>0.05). Females reached the training criteria in fewer sessions than males (sessions to reach criteria were 61 and 35 for males and females, respectively; sex effect, F_1,12_ = 6.45, *p*<0.05). However, no genotype×sex interaction was found (F_1,12_ = 1.25, *p*>0.05). Of a total of 21 animals, 5 failed to reach the training criteria for reliable morphine discrimination in 40 training session (three female GluA1+/+ mice and one male and one female GluA1−/− mouse) and were therefore excluded from the analyses.

**Figure 4:**
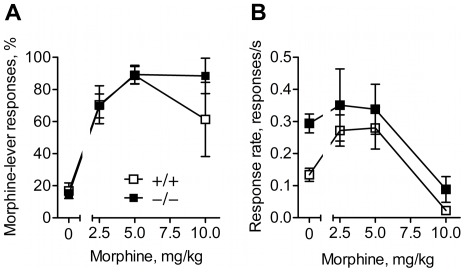
Unaltered detection of opioid stimulus in GluA1−/− mice. Mice were trained to discriminate 5 mg/kg morphine from saline and then tested for various doses of morphine in a 20-min drug discrimination test session. (**A**) Dose-response curves for discriminative stimulus effects of morphine; data show morphine-appropriate lever responses expressed as percentage of total responses on both levers. (**B**) Dose-response curves for response rate-lowering effects of morphine during the dose-response tests. Data are shown as means ± SEM, *n* = 5–6.

After the discrimination training, dose-response tests were performed. Figure 4A shows the morphine dose-response function with morphine-appropriate responding during the 20-min session. There was a significant dose effect (F_3,21_ = 14.59, *p*<0.001) with higher morphine doses producing higher morphine-appropriate responding. However, no genotype (F_1,7_ = 0.72, *p*>0.05) or sex (F_1,7_ = 0.07, *p*>0.05) effects and no interactions (*p*'s>0.05) were found. Figure 4B shows that response rates declined with increasing morphine dose (dose effect, F_3,21_ = 6.51, *p*<0.01). There were no significant effects of sex (F_1,7_ = 0.01, *p*>0.05) or genotype (F_1,7_ = 1.88, *p*>0.05) and no significant interactions (*p*'s>0.05). Statistical analysis for both the morphine-appropriate responding and the response rate data were also performed without the data for the highest morphine testing dose (10 mg/kg) which tended to produce non-responding behavior in both genotypes. These analyses, however, revealed no significant genotype differences (data not shown).

### AMPA/NMDA ratio of VTA dopamine neurons

**Figure 5:**
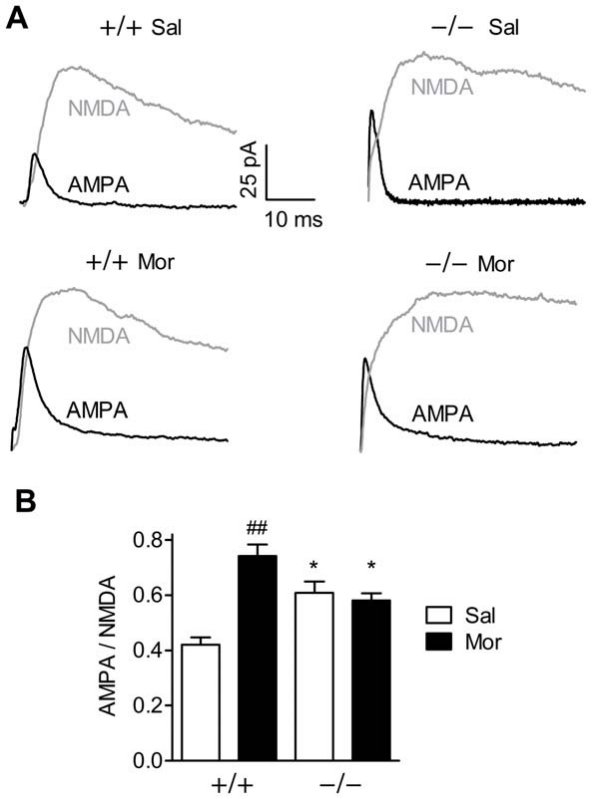
Lack of morphine-induced increase in the ventral tegmental area dopamine neuron AMPA/NMDA ratios in GluA1−/− mice. A single morphine (10 mg/kg) or saline injection was administered one day before *ex vivo* electrophysiological recordings from slices. (**A**) Representative traces displaying AMPA and NMDA receptor-mediated current components. (**B**) Quantification of the electrophysiological data, which is represented as means ± SEM, *n* = 6–11. ^*^ *p*<0.05, between the genotypes; ^##^ *p*<0.01, compared to the corresponding saline control, Bonferroni test.

By measuring AMPA/NMDA ratio, we wanted to compare the strength of glutamate synapses of VTA DA cells 24 h after a single *in vivo* injection of morphine (10 mg/kg) to young GluA1−/−and GluA1+/+ mice. The mouse lines responded differently to the treatment (genotype×drug interaction, F_1,27_ = 20.32, *p*<0.001). In GluA1+/+ mice, morphine strongly increased AMPA/NMDA ratio (drug effect, F_1,27_ = 14.14, *p*<0.01), as shown in Figure 5. However, no such increase was detected in GluA1−/− mice. However, after saline injection, the baseline AMPA/NMDA ratio was higher in GluA1−/− than in GluA1+/+ mice (*p*<0.05).

To determine the basal locomotor behavior of young (P20–26, the age also used for electrophysiology) GluA1−/− and GluA1+/+ mice, a 30-min locomotor activity test was run in exactly the same conditions as the place conditioning described above. As in the adult mice, locomotor activity of the young GluA1−/− mice (115.0±6.3 m) was greater than that of GluA1+/+ mice (48.2±2.6 m) (genotype effect, F_1,31_ = 98.61, *p*<0.001). Young GluA1−/− mice were, thus, moving 2.40 times more than GluA1+/+ mice, the ratio being almost identical to that in adults (2.43 times more) treated with saline during the first conditioning trial. No significant effect of sex or genotype×sex interaction was found (*p*>0.05).

### μ-Opioid receptor G-protein activation

**Figure 6:**
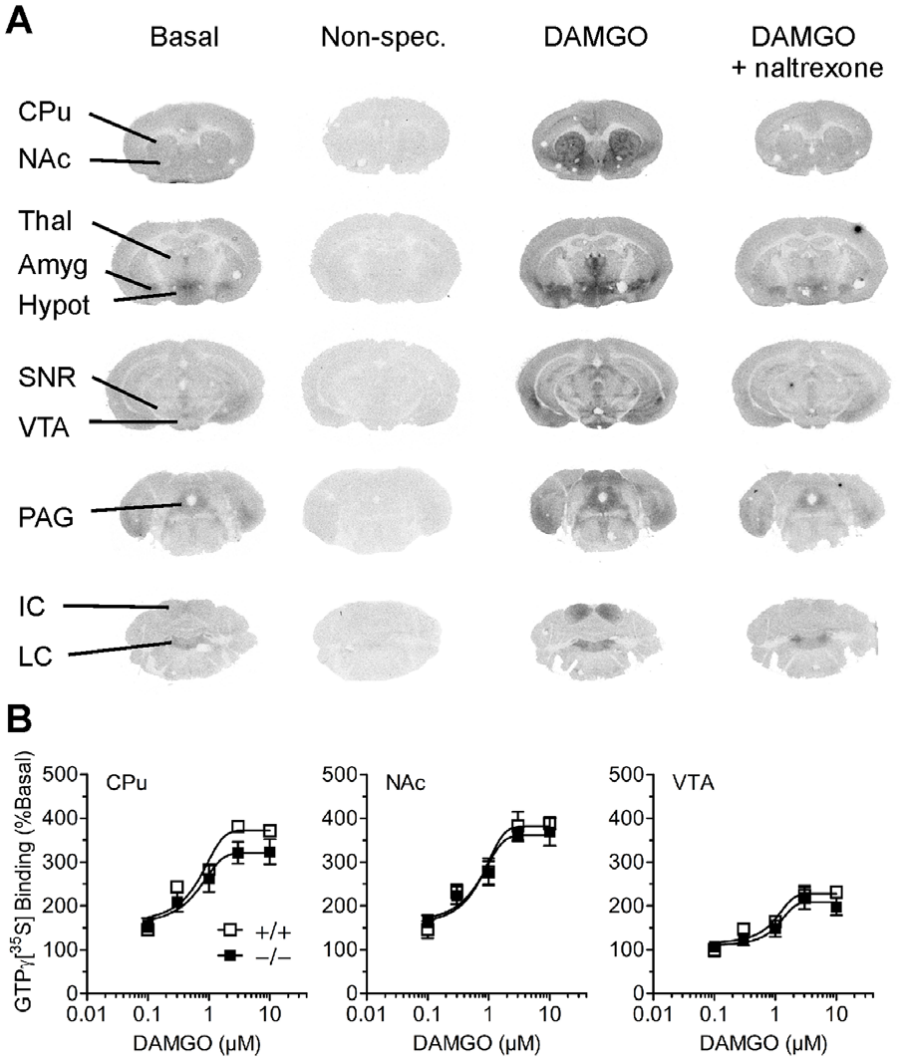
Unchanged μ-opioid receptor function in GluA1−/− mice as shown by DAMGO-stimulated GTPγ[^35^S] autoradiography. (**A**) Representative autoradiographs from a control GluA1+/+ mouse showing GTPγ[^35^S] binding in various brain regions. The figure shows basal binding, non-specific (Non-spec.) binding in the presence of 10 µM cold GTPγS and specific 10 µM DAMGO-stimulated binding with and without the opioid receptor antagonist naltrexone (10 µM). (**B**) Dose-response curves of DAMGO-stimulated GTPγ[^35^S] binding in the caudate-putamen, nucleus accumbens and ventral tegmental area. The data are means ± SEM, *n* = 5–7. See Table 1 for EC_50_ and maximal stimulation values for DAMGO. Amyg, amygdala; CPu, caudate-putamen; Hypot, hypothalamus; IC, inferior colliculus; LC, locus coeruleus; NAc, nucleus accumbens; PAG, periaqueductal gray; SNR, substantia nigra reticulata; Thal, thalamus; VTA, ventral tegmental area.

By using the GTPγ[^35^S] autoradiographic analysis, we aimed to assess the brain regional μ-opioid receptor signal transduction at the G-protein level in GluA1−/− and GluA1+/+ mice. Representative autoradiographs are depicted in Figure 6. Basal and non-specific bindings were equal in both genotypes and sexes across all brain regions (data not shown). In DAMGO-stimulated regional EC_50_-values (Table 1), no statistically significant differences were detected between genotypes or sexes (*p*'s>0.05). However, the EC_50_ value was higher in the VTA than in the amygdala, caudate-putamen, hypothalamus, locus coeruleus, nucleus accumbens and thalamus (ANOVA for region F_9,86_ = 3.71, *p*<0.01, followed by Bonferroni test) suggesting lower potency for DAMGO in the VTA. In maximal stimulation (Table 1), no differences were observed between genotypes or sexes in any of the brain regions studied (*p*'s>0.05). GTPγ[^35^S] binding stimulated by 10 µM morphine was similar between GluA1−/− and GluA1+/+ mice in all brain regions studied (data not shown).

**Table 1:** EC_50_ and maximal stimulation values for DAMGO stimulation of GTPγ[^35^S] binding in various brain regions of GluA1+/+ and GluA1−/− mice.

Region	EC_50_		Maximal stimulation	
	GluA1+/+	GluA1−/−	GluA1+/+	GluA1−/−
Caudate-putamen	0.45±0.07	0.44±0.09	394±18	337±23
Nucleus accumbens	0.65±0.17	0.45±0.11	414±8	383±28
Thalamus	0.41±0.07	0.43±0.09	293±14	294±21
Amygdala	0.41±0.07	0.29±0.11	235±13	245±15
Hypothalamus	0.47±0.12	0.26±0.06	183±8	197±8
Substantia nigra reticulata	0.91±0.32	0.69±0.15	181±19	176±7
Ventral tegmental area	1.20±0.29	1.35±0.49	254±12	224±22
Periaqueductal gray	0.87±0.25	0.38±0.09	176±14	198±10
Inferior colliculus	1.03±0.31	0.63±0.17	276±18	290±21
Locus coeruleus	0.59±0.33	0.38±0.12	174±15	177±10

Values are mean ± SEM, n = 5–7, in µM for EC_50_ values and as percentage of basal for maximal stimulation.

## Discussion

In the present study, we found that morphine state-dependency was altered in the mouse line lacking the GluA1 subunit of the AMPA-type glutamate receptor. State-dependency was probed in three conditions: Firstly, when adult GluA1+/+ mice were tested in a state (Mor10-state) equal to the conditioning state, they expressed a significant place preference. However, GluA1−/− mice failed to show place preference in this condition. Secondly, both lines conditioned with 20 mg/kg morphine, and tested in Mor10-state (now mice received half of the conditioning dose and thus were in a different state) failed to express place preference. Thirdly, some expression of morphine-induced place preference was detected in both mouse lines after being conditioned with morphine (10 or 20 mg/kg) and tested in Sal-state. These data indicate that a testing dose equal to conditioning dose (i.e. state-dependency) produces the greatest expression of place preference, also demonstrated by others in the place preference paradigm [24], [25]. Importantly, these data suggest a deficit in opioid state-dependency in the GluA1−/− mouse line.

Studies elaborating state-dependency with drug-induced place conditioning are sparse (see [26]), but some other methods have been used, e.g. operant food-reinforced system [5] and passive avoidance [27]. The data presented here show similar pattern of state-dependency as these previous observations using other methods, that is, the greatest responding is acquired in the same drug state while out-of-state responding is lower, but not non-existent [5], [27]. Morphine-induced state-dependency has been shown to rely on selectivity of opioid receptors, and on various other neurotransmitter systems, such as the DA system, in the brain [5], [27], [28]. Our data point to a significant role of AMPA-type glutamate receptors in state-dependency.

Detection of opioid stimulus was found to be intact in GluA1−/− mice, since these mice were able to discriminate between the levers associated with morphine and saline when different doses of morphine were administered at the drug discrimination test. The GluA1−/− mice learned the food-reinforced lever-pressing similarly to their wildtype controls, thus providing a stable platform to study drug discrimination and confirming the previous data that GluA1 subunit is not critical in learning the simple food-reinforced lever-pressing task [14]. Unchanged perception of morphine effects by these mice was indicated by the unchanged dose-response of morphine-appropriate responding. To our knowledge, these are the first morphine discrimination studies reported for mice. Importantly, most opioid-related mechanisms and processes that have been studied, have been unaltered in the GluA1−/− mice. They have unaltered pharmacodynamics evidenced by unchanged μ-opioid receptor signaling at the G-protein level in various brain regions (the present study), morphine-induced sensitization and morphine-induced analgesia [16] and pharmacokinetics evidenced by unchanged morphine concentrations in blood and brain tissue after acute administration [16]. Thus, the target systems for morphine appear to be normally functional in the GluA1−/− mice.

Patch-clamp recordings, performed in young mice (P20–26), revealed that the VTA DA neurons of the GluA1−/− mice failed to show glutamate receptor neuroplasticity in the same manner as those of GluA1+/+ mice after a single morphine administration. This deficient neuroplasticity has been observed in the GluA1−/− mice also after cocaine administration [13]. The elevation of AMPA/NMDA receptor current ratio is considered to represent NMDA receptor-dependent long-term potentiation (LTP) in the VTA [20], [29]. Activation of VTA DA neurons by the drugs of abuse elevates the level of GluA1 subunit in the VTA [30], [31]. According to the current hypothesis, the activation of VTA DA neurons leads to a fast insertion of calcium-permeable non-GluA2 subunits (GluA1, GluA3 or GluA4) to their afferent synapses, which provide an additional source of calcium, leading to LTP [32]–[34]. Impaired LTP may be more general in the GluA1−/− mice, as LTP is also absent in the hippocampal CA1 area of these mice [10]. The baseline AMPA/NMDA ratio of the GluA1−/− mice was elevated, in agreement with Dong et al. [13]. The mechanism of this alteration is not known and interestingly, genome-wide expression profiling has revealed no upregulation of GluA3 (or ectopic GluA4) expression, but apparently compensatory upregulation of calcium channels in the GluA1−/− mice [35]. It should be noted that, in addition to changes in excitatory inputs to VTA DA neurons, the global knockout of GluA1 subunit in GluA1−/− mice might have induced some unknown changes during nervous system development. Nevertheless, the maximal elevation in the AMPA/NMDA ratio was unlikely reached in GluA1−/− mice since the morphine-induced AMPA/NMDA ratio was higher in GluA1+/+ mice than in GluA1−/− mice.

Besides state-dependency, the current experiments also enabled to examine reward behavior in the absence of GluA1 subunit-containing AMPA receptors. No deficit in the morphine-induced place preference in GluA1−/− mice in the undrugged test situation (Sal-state) was found, suggesting that these mice are capable of learning the CS-US association and express normal place preference. Results from earlier conditioned place preference studies with GluA1−/− mice using cocaine are inconsistent, reporting either a deficit [13] or no deficit [36], [37] in the expression of cocaine-conditioned place preference. However, these results are dependent upon the design of the experiment. The deficit in place preference was only observed when using a biased apparatus with non-counterbalanced (biased) subject assignment [13], which both are factors that complicate the interpretation of the place preference data [38]. This precaution is important especially in the case of morphine that can exert anxiolytic effects [39]. The present experiments were designed to exclude the apparatus bias over the conditioning phase by inclusion of saline-saline control animals. In agreement with our findings, the pharmacological approach using AMPA receptor antagonists suggests that these receptors are dispensable in drug-induced conditioned place preference, as long as the ligands are selective to the AMPA receptors and do not affect the NMDA receptor functions [37], [40], [41].

The neurobiological control of locomotor activity, either spontaneous or that initiated by the drugs of abuse, has previously been suggested to arise, at least partly, from the mesolimbic dopamine system [42]. The GluA1−/− mice show a robust endophenotype of novelty-induced hyperactivity in the present data and in [16], [43], an observation that could be due to the modified glutamate receptor activation in the VTA DA neurons. Thus, either the baseline elevation in AMPA/NMDA ratio or the inability to adapt to exogenous stimuli due to the static AMPA/NMDA ratio of GluA1−/− mice might contribute to the hyperactive phenotype. However, the VTA DA cells are unlikely causes for the hyperactive phenotype, since (i) GluA1−/− mice show no difference in activation of the VTA DA cells in response to novelty in the similar experimental setting as used here [44], (ii) no change in locomotor activity was observed despite the sustained elevation of AMPA/NMDA ratio in wild-type mice pretreated one day earlier with drugs of abuse [17], [45], and (iii) a mouse line lacking GluA1 in the VTA DA cells shows both unchanged baseline locomotor activity and AMPA/NMDA ratio [36].

The hyperactivity of GluA1−/− mice could have interfered with the expression of conditioned place preference. Unfortunately, locomotor activity is seldom measured or reported in conditioned place preference studies. In the study by Vezina and Stewart [23], in which hyperactivity was suggested to mask the expression of place preference, the testing chamber was greater in size than that used during the conditioning phase. This difference may have predisposed animals to enhanced drive for exploring the testing chamber. Thus, increased exploration due to changed environment might have caused decreased expression of conditioned place preference, not the increased locomotor activity. In the present study, we co-measured locomotor activity during the place preference test trials. Although GluA1−/− mice had higher locomotor activity than the GluA1+/+ mice, no correlation between the locomotor activity and place preference was found in our data. Locomotor activity of GluA1+/+ mice tested in Mor10-state was equal between groups of mice that were conditioned with either 10 mg/kg or 20 mg/kg morphine, but only 10 mg/kg produced high expression of CPP. It cannot be fully excluded that increased locomotor activity *per se* affected the expression of CPP, but this is unlikely, since Mead and Stephens [37] have reported the GluA1−/− mice to display both normal cocaine-induced place preference and increased locomotor activity during the preference test trial.

Morphine, administered in the first conditioning trial, did not enhance locomotor activity of GluA1−/− mice, whereas it enhanced the activity of GluA1+/+ mice. GluA1−/− mice displayed increased habituation compared to GluA1+/+ mice. It is possible that habituation to conditioning cages might have masked the locomotor-activating effect of the first morphine dose. In support to this, the lack of the morphine effect was transient and in the second conditioning trial the morphine effect was observed also in GluA1−/− mice. It is also possible that the lack of AMPA receptor adaptation in GluA1−/− mice might have increased the sedative effect of the first dose of morphine and thereby prevented opioid-induced hyperactivity.

Since morphine-induced psychomotor sensitization and conditioned place preference were observed in the GluA1−/− mice, these behaviors can be dissociated from the glutamate receptor neuroplasticity in the VTA DA neurons. This is supported by the results from a transgenic mouse line with GluA1 subunits lacking from the DA neurons, which show both unaltered place preference and sensitization in the absence of drug-induced plasticity in VTA DA cells [36]. However, it is possible that drug-induced glutamate plasticity in VTA DA neurons, as expressed by the elevation in AMPA/NMDA ratio, is linked to state-dependency, since the drug-state seems not to be detectable as synaptic plasticity of the VTA DA neurons in GluA1-deficient mice.

In summary, GluA1−/− animals were impaired in expressing conditioned place preference when tested in drugged state. Importance of state-dependency for neurobiology of addiction has previously been suggested [1], and the present data are important in suggesting a role for GluA1 subunit-containing AMPA receptors in morphine state-dependency. AMPA receptor-mediated plasticity in response to the drugs of abuse has previously been suggested to underlie at least the initial steps in the development of drug dependence and addiction [12], and our data further strengthen the significance of AMPA receptors in these processes.
